# Metabolic syndrome and its predictors in an urban population in Kenya: A cross sectional study

**DOI:** 10.1186/s12902-017-0188-0

**Published:** 2017-07-04

**Authors:** Geoffrey Omuse, Daniel Maina, Mariza Hoffman, Jane Mwangi, Caroline Wambua, Elizabeth Kagotho, Angela Amayo, Peter Ojwang, Zulfiqarali Premji, Kiyoshi Ichihara, Rajiv Erasmus

**Affiliations:** 1 Department of Pathology, Aga Khan University Hospital Nairobi, P.O. Box 30270-00100, Nairobi, Kenya; 2 Division of Chemical Pathology, Department of Pathology, Stellenbosch University, P.O. Box 19113, Tygerberg Hospital, Cape Town, South Africa; 3 PathCare Kenya Ltd., P.O. Box 12560-00606, Nairobi, Kenya; 4 Department of Human Pathology, University of Nairobi, P.O. Box 19676-00200, Nairobi, Kenya; 5 Department of Pathology, Maseno University, P.O. Box Private Bag, Maseno, Kenya; 6 Formerly of Muhimbili University of Health and Allied Sciences, Dar es Salaam, Tanzania; 7 Graduate School of Medicine, Faculty of Health Sciences, Yamaguchi University, Minami-Kogushi 1-1-1, Ube, 755-8505 Japan

**Keywords:** Metabolic syndrome, Waist circumference, Visceral adiposity, Cardiovascular risk, Kenya, Africa

## Abstract

**Background:**

The metabolic syndrome (MetS) is a clustering of interrelated risk factors which doubles the risk of cardio-vascular disease (CVD) in 5–10 years and increases the risk of type 2 diabetes 5 fold. The identification of modifiable CVD risk factors and predictors of MetS in an otherwise healthy population is necessary in order to identify individuals who may benefit from early interventions. We sought to determine the prevalence of MetS as defined by the harmonized criteria and its predictors in subjectively healthy black Africans from various urban centres in Kenya.

**Method:**

We used data collected from healthy black Africans in Kenya as part of a global study on establishing reference intervals for common laboratory tests. We determined the prevalence of MetS and its components using the 2009 harmonized criterion. Receiver operator characteristic (ROC) curve analysis was used to determine the area under the curves (AUC) for various predictors of MetS. Youden index was used to determine optimum cut-offs for quantitative measurements such as waist circumference (WC).

**Results:**

A total of 528 participants were included in the analysis. The prevalence of MetS was 25.6% (95% CI: 22.0%–29.5%). Among the surrogate markers of visceral adiposity, lipid accumulation product was the best predictor of MetS with an AUC of 0.880 while triglyceride was the best predictor among the lipid parameters with an AUC of 0.816 for all participants. The optimal WC cut-off for diagnosing MetS was 94 cm and 86 cm respectively for males and females.

**Conclusions:**

The prevalence of MetS was high for a healthy population highlighting the fact that one can be physically healthy but have metabolic derangements indicative of an increased CVD risk. This is likely to result in an increase in the cases of CVD and type 2 diabetes in Kenya if interventions are not put in place to reverse this trend. We have also demonstrated the inappropriateness of the WC cut-off of 80 cm for black African women in Kenya when defining MetS and recommend adoption of 86 cm.

## Background

The non-communicable disease (NCD) burden is expected to increase globally by 17% and by 27% in the African region in the next 10 years. NCDs are projected to overtake communicable diseases as the major cause of morbidity in sub Saharan Africa by the year 2030 [1]. Smoking, hypertension, abdominal obesity, diabetes mellitus and elevated Apolipoprotein B/A-1 ratio have been shown to account for up to 90% of the risk for a first myocardial infarction in Africa according to the INTERHEART study [2]. Unfortunately, the prevalence of these risk factors continues to increase as urbanization takes root in the African continent [3].

The metabolic syndrome (MetS) is a clustering of interrelated risk factors which doubles the risk of cardio-vascular disease (CVD) in 5–10 years and increases the risk of type 2 diabetes 5 fold [4]. The identification of modifiable CVD risk factors and predictors of MetS in an otherwise healthy population is necessary in order to identify individuals who may benefit from early interventions. It has been shown that subjectively healthy individuals may have biochemical abnormalities in keeping with the presence of MetS [5]. A study carried out in the US showed that 23.5% of normal-weight adults were metabolically abnormal and conversely, 51.3% of overweight adults and 31.7% of obese adults were metabolically healthy [6]. Metabolic derangements may be an early indicator of increased CVD risk even in normal weight or subjectively healthy individuals. Initial results of reference interval (RI) studies carried out in Japan, China, Turkey, Saudi Arabia and USA have shown that the levels of fasting plasma glucose (FPG), triglycerides (TGs), alanine aminotransferase (ALT) and gamma-glutamyl transferase (GGT) increase while high density lipoprotein cholesterol (HDL-C) decreases as body mass index (BMI) increases. This pattern of reduced HDL-C, increased TG and FPG suggests the presence of MetS in some of the reference individuals recruited despite adherence to a strict inclusion criteria designed to exclude unhealthy individuals. This has raised the question whether RIs should be further stratified according to BMI for those analytes where it is a major source of variation [7].

Whereas increasing levels of waist circumference (WC) and BMI have been associated with increased CVD risk, there is a continuous search for robust predictors of MetS. Both BMI and WC do not distinguish between visceral adipose tissue (VAT) and subcutaneous adipose tissue (SAT). VAT plays a significant role in the pathogenesis of CVD due to its association with insulin resistance and increased levels of VAT has been associated with increased cardio-metabolic risk and coronary artery calcification regardless of BMI [8, 9]. One of the emerging surrogate markers of visceral adiposity and predictor of MetS yet to be extensively studied in a black African population is the lipid accumulation product (LAP), a parameter whose calculation is based on WC and serum TG levels [10]. LAP has been shown to have a strong association with the presence of MetS in healthy adults, an accurate predictor of MetS in those aged 50 years and above, as well as an association with diabetes in studies carried out in Europe and Asia [11–14].

Visceral adiposity index (VAI) is another emerging parameter that indirectly expresses visceral fat function and has been found to be independently associated with coronary heart disease, myocardial infarction, transient ischemic attack and ischemic stroke. The calculation of VAI is based on WC, BMI, triglycerides, and HDL cholesterol and hence includes both physical and metabolic parameters [15]. In China, VAI was shown to be positively associated with type 2 diabetes and was a better predictor of diabetes than BMI, WC and waist to height ratio [16]. VAI was derived from an Italian cohort largely comprising of Caucasians and hence needs validation in a black African population.

Hypertriglyceridaemia and low HDL-C are the only lipid parameters included in the harmonized MetS criterion [4]. There is controversy as to the specific role elevated TGs play in atherogenesis. Whereas increased TGs have been associated with increased risk of CVD, it is thought that they are a surrogate marker for cholesterol remnants which play a direct role in atheromatous plaque formation [17]. Varbo et al. demonstrated in a Danish population that non-fasting cholesterol remnants are a risk factor for ischaemic heart disease independent of HDL-C levels with an increase of 1 mmol/L associated with a 2.8 fold increase in risk [18]. The utility of cholesterol remnants in determination of CVD risk or MetS in healthy black Africans is unknown.

The prevalence of MetS in any population is influenced by many factors including the specific criteria adopted to define it. For example, in a rural population in Ghana, the overall prevalence of MetS defined by the International Diabetic Federation (IDF) and the National Cholesterol Education Programme Adult Treatment Panel III (ATP III) criteria was 35.9% and 15.0% respectively [19]. In 2009, a harmonized criterion for diagnosing MetS was developed whose components included abdominal obesity, dyslipidemia, hyperglycemia, and hypertension [4]. Using the harmonized definition, the prevalence of MetS in an urban population in Kenya was reported as 34.6% with the prevalence being significantly higher in women at 40.2%. However, this study was only limited to one constituency in the capital city of Nairobi which limits the generalizability of the results despite the use of random sampling when identifying participants [20]. Furthermore, this study didn’t explore the utility of LAP and VAI as predictors of MetS in a black African population.

We sought to determine the prevalence of MetS as defined by the harmonized criteria in subjectively healthy black Africans from various urban centres in Kenya. We also determined the ability of various lipid parameters, LAP, VAI, WC and BMI to predict the presence of MetS.

## Methods

We used data from 533 healthy black Africans participating in a global RI study in Kenya. This study is part of an initiative by the Committee of Reference Intervals and Decision Limits (C-RIDL) under the auspices of the International Federation of Clinical Chemistry (IFCC). Kenya is one of 3 participating countries in Africa, the other ones being Nigeria and South Africa. Majority of recruited participants in Kenya were urban dwellers from the capital city Nairobi and its environs, Kisii County in the western part of Kenya and Nakuru town based in the Great Rift valley. Recruitment was mainly done by seeking adult volunteers aged 18–65 years of age through use of posters in public areas including churches, universities, colleges, hospitals and companies. Social media and word of mouth were also used. This was done between January and October 2015 and written informed consent was sought from each participant after giving a written and verbal explanation of the study.

### Inclusion criteria

Inclusion was limited to healthy adults 18–65 years of age and was stratified into 4 age groups: 18–29, 30–39, 40–49 and 50–65 years with a similar distribution of males and females in each age strata.

### Exclusion criteria

Exclusion criteria included participants with a BMI greater than 35 kg/m^2^, consumption of ethanol greater than or equal to 70 g per day [equivalent to 5 alcoholic drinks], smoking more than 20 tobacco cigarettes per day, taking regular medication for a chronic disease (diabetes mellitus, hypertension, hyperlipidemia, allergic disorders, depression), recent (less than 15 days) recovery from acute illness, injury or surgery requiring hospitalization, known carrier state of hepatitis B, hepatitis C or human Immunodeficiency virus, pregnant or within one year after childbirth. Individuals with any chronic disease were excluded except for the age group 50–65 years where those with well controlled hypertension were recruited. Towards the end of the study, a few individuals with a BMI greater than 35 kg/m^2^ but less than 40 kg/m^2^ were recruited due to difficulties in recruiting healthy participants in the older age groups. The exclusion criteria were defined in the questionnaire filled by the participants and those who were excluded didn’t have blood samples taken.

### Measurements

All participants had measurements of blood pressure (BP), WC and body mass index (BMI) taken. A single measurement of BP was performed in a sitting position after at least 15 min of rest using a calibrated OMRON M3 automated BP monitor (Omron Healthcare, Kyoto, Japan) that uses an upper arm cuff. A repeat BP measurement was done if the initial reading was consistent with hypertension. If the initial and second reading were in agreement then the first reading was adopted. If there was a discrepancy in terms of BP status then a third reading was done as a tie breaker. WC was measured using a tape measure over light clothing at the level of the umbilical cord to ensure a consistent reference point while BMI was automatically calculated after measuring the participants’ height and weight using a Seca 703 weighing scale digital column with height meter scale (Seca, Hamburg, Germany). On average, measuring WC over light clothing increased the measurement by approximately 1 cm which was subsequently deducted. Height was measured after removal of shoes to the nearest 0.5 cm, WC to the nearest 1 cm and weight to the nearest 0.1 kg after removal of shoes and bulky clothing.

### Sample handling

All participant samples were collected after obtaining informed consent and filling of a questionnaire. All participants had an overnight fast as per the study protocol. Samples were collected and centrifuged within 4 h after collection and stored at −80°c until shipment on dry ice to the reference laboratory in South Africa. All the sample analysis for the biochemistries and immunoassays were performed at the Pathcare reference laboratory in Cape Town, South Africa which is an International Organization for Standardization (ISO) 15189 accredited laboratory. Thawing was only done once before sample analysis. As part of the RI study, all participating laboratories used a common panel of sera with assigned values to ensure accuracy of reported results and alignment of values if any biases were identified. All the listed tests in Table 1 were carried out on a Beckman Coulter AU5800. The analytical methods for the various tests are shown in Table 1.

**Table 1:** List of tests, methodologies and coefficient of variation

Test	Method	Between run CV
UA	Enzymatic colour	0.1%
TC	Enzymatic colour calibrated to CDC Reference Method (Abell-Kendall)	0.3%
LDL-C	Enzymatic colour calibrated to US CDC	0.4%
TGs	Enzymatic colour	0.4%
HDL-C	Enzymatic colour calibrated to US CDC	0.3%
Glucose	Enzymatic UV test (hexokinase method)	0.1%
hsCRP	Immuno-turbidimetric	0.8%
ALP	Kinetic colour IFCC	0.1%
ALT	Kinetic UV IFCC	0.8%
AST	Kinetic UV IFCC	0.4%
GGT	Kinetic colour IFCC	0.4%

*Abbreviations ALP* Alkaline phosphatase, *ALT* Alanine aminotransferase, *AST* Aspartate aminotransferase, *CDC* Centre of Disease Control, *CV* Coefficient of variation, *GGT* Gama glutamyl transferase, *HDL-C* High density lipoprotein cholesterol, *hsCRP* Highly sensitive C-reactive protein, *IFCC* International Federation of Clinical Chemistry, *LDL-C* Low density lipoprotein cholesterol, *TGs* Triglycerides, *UA* Uric acid, *US* United States

### MetS diagnosis

The 2009 harmonized definition was used to diagnose MetS which requires the presence of any 3 of the following: increased WC (men: ≥ 94 cm, women: ≥ 80 cm), low HDL-C (men: <40 mg/dl (1 mmol/l), women: <50 mg/dl (1.3 mmol/l)), hypertriglyceredimia ≥150 mg/dl (1.7 mmol/l), elevated BP (systolic BP ≥ 130 mmHg and/or diastolic ≥85 mmHg or drug treatment for hypertension) and elevated blood sugar (FPG ≥ 100 mg/dl (5.6 mmol/l) or diabetes mellitus [4].

### CVD risk calculation

The 10 year CVD risk was calculated using the Framingham risk calculation [21]. Family history of CVD was not included as this information wasn’t captured in the study questionnaire.

### LAP calculation

LAP was calculated as (WC [cm] − 65) × (TG [mmol/L]) for males, and (WC [cm] − 58) × (TG [mmol/L]) for females [10].

### VAI calculation

VAI was calculated as (WC[cm]/(39.68 + (1.88 x BMI))) x (TG/1.03) x (1.31/HDL-C [mmol/L]) for males and (WC[cm]/(36.58 + (1.89 x BMI))) x (TG/0.81) x (1.52/HDL-C [mmol/L]) for females [15].

### Non HDL-C

Non HDL-C was calculated as fasting total cholesterol minus HDL-C.

### Cholesterol remnants

Cholesterol remnants were calculated as fasting total cholesterol minus HDL-C minus LDL-C.

### Statistical analysis

The prevalence of MetS was presented as a percentage with 95% confidence intervals (CI). Descriptive statistics for continuous variables were presented as medians with interquartile ranges (IQRs). The Mann Whitney U test was used to compare the distribution pattern of continuous variables between males and females and those with and without MetS. Where the distribution patterns were similar, medians were compared using the independent samples median test. Mean ranks were compared where the distribution patterns were not similar. Binary logistic regression was used to determine the association between cardio-metabolic risk factors and MetS presence. Adjusted odds ratios (ORs) were subsequently determined in a model that excluded the MetS components. For hs-CRP, uric acid and GGT, the median value was used as a cut-off to classify individuals as having increased or normal levels. Binary logistic regression was also used to determine the strength of association between MetS and CVD risk. Chi-square was used to compare cardio-metabolic risk factors between male and female participants as well as between individuals with a BMI < 25 kg/m^2^ and those with BMIs ≥25 kg/m^2^. A *p*-value < 0.05 was considered statistically significant. Receiver operator characteristic (ROC) curves were created and area under the curve (AUC) determined in order to compare lipid parameters, LAP, VAI, WC and BMI as predictors of MetS. An AUC ≥ 0.90 was considered excellent; 0.80–0.90, good; 0.70–0.80, fair; and 0.70–0.50, poor test performance. The Youden’s index was calculated and used to determine the cut-offs that gave the best combination of sensitivity and specificity. Statistical analysis was carried out using IBM SPSS Statistics for Windows, Version 20.0 (Armonk, NY: IBM Corp).

## Results

A total of 533 participants met the inclusion criteria and subsequently had samples collected and analysed. However, 5 participants didn’t have FPG results due to sample insufficiency and were excluded from this analysis leaving 528 participants, 255 (48.3%) males and 273 (51.7%) females. BMI was higher in female participants while WC and BP were higher in males. The difference in total cholesterol (TC) between male and female participants was not statistically significant but TGs were significantly higher in males and HDL-C lower in females. FPG levels and liver enzymes were higher in male participants as shown in Table 2. Only 2 (0.7%) women were smokers compared to 13 (5.1%) men, a difference that was statistically significant (*p* = 0.003).

**Table 2:** Descriptive characteristics of participants

	Male (*n* = 255)		Female (*n* = 273)		Total (*n* = 528)		Male vs Female
	Median (IQR)	Min-Max	Median (IQR)	Min-Max	Median (IQR)	Min-Max	*p*-value
Age (years)	38 (19)	20–65	39 (21)	18–64	39 (20)	18–65	0.986*
BMI (kg/m^2^)	24.87 (5.66)	16.29–34.94	26.10 (6.25)	17.10–38.05	25.46 (5.96)	16.29–38.05	0.000^†^
LAP	29.52 (40.95)	0.00–388.87	23.97 (29.69)	3.30–205.54	26.94 (35.96)	0.00–388.87	0.055*
VAI	1.51 (1.45)	0.26–21.66	1.35 (1.13)	0.42–20.42	1.43 (1.22)	0.26–21.66	0.163*
WC (cm)	90 (15)	65–124	86 (16)	64–115	89 (17)	64–124	0.000^†^
SBP (mmHg)	127 (18)	94–167	118 (20)	77–194	123.5 (20)	77–194	0.000^†^
DBP (mmHg)	81 (12)	56–101	79 (14)	57–112	80 (14)	56–112	0.003^†^
FPG (mmol/L)	4.9 (0.8)	3.0–15.6	4.8 (0.7)	3.3–19.5	4.9 (0.8)	3.0–19.5	0.022^†^
TC (mmol/L)	4.7 (1.2)	2.3–8.2	4.6 (1.2)	2.6–7.7	4.6 (1.1)	2.3–8.2	0.732*
HDL-C (mmol/L)	1.1 (0.3)	0.5–2.0	1.2 (0.3)	0.3–2.4	1.1 (0.3)	0.3–2.4	0.000^†^
cLDL-C (mmol/L)	2.9 (1.1)	1.0–5.8	2.9 (1.0)	1.1–5.4	2.9 (1.0)	1.0–5.8	0.650*
mLDL-C (mmol/L)	2.9 (1.1)	0.9–5.8	2.8 (1.0)	1.3–5.2	2.8 (1.1)	0.9–5.8	0.141*
TG (mmol/L)	1.2 (0.90)	0.32–10.51	0.9 (0.61)	0.33–4.78	1.05 (0.75)	0.32–10.51	0.000^†^
UA (mmol/L)	0.35 (0.10)	0.18–0.64	0.27 (0.08)	0.13–0.47	0.31 (0.11)	0.13–0.64	0.000^†^
ALP (U/L)	86 (35)	34–179	84 (38)	31–191	85 (37)	31–191	0.497*
ALT (U/L)	21 (14)	8–96	14 (9)	6–123	18 (11)	6–123	0.000^†^
AST (U/L	25 (8)	14–73	21 (6)	11–102	23 (7)	11–102	0.000^†^
GGT (U/L)	31 (24)	9–701	21 (12)	7–211	25 (19)	7–701	0.000^†^
hsCRP (mg/L)	0.99 (2.05)	0.20–36.25	1.65 (3.19)	0.20–60.75	1.31 (2.63)	0.20–60.80	0.000^†^

*Abbreviations ALP* Alkaline phosphatase, *ALT* Alanine aminotransferase, *AST* Aspartate aminotransferase, *BMI* Body Mass Index, *BP* Blood pressure, *CVD* Cardiovascular disease, *DBP* Diastolic blood pressure, *F* Female, *FPG* Fasting plasma glucose, *GGT* Gama glutamyl transferase, *HDL-C* High density lipoprotein cholesterol, *hsCRP* Highly sensitive C-reactive protein, *LAP* Lipid accumulation product, *cLDL-C* calculated low density lipoprotein cholesterol, *mLDL-C* measured low density lipoprotein cholesterol, *M-Male* SBP-Systolic blood pressure, *TGs* Triglycerides, *VAI* Visceral adiposity index, *UA* Uric acid, *WC* Waist circumference

*comparison of medians

^†^comparison of mean ranks

The overall prevalence of MetS was 25.6% (95% CI: 22.0%–29.5%). The prevalence increased with increase in age and BMI. The most prevalent component of the MetS was increased WC which was present in 294 (55.7%) participants while the least prevalent was elevated FPG which was found in 83 (15.7%) participants. Having GGT or UA values above the median more than doubled the odds of having MetS as shown in Table 3. Only 2 out of 135 (1.5%) participants with MetS were smokers compared to 13 out of 393 (3.3%) with no MetS (*p* = 0.375). Those who consumed alcohol were 26.7% (36/135) and 34.6% (136/393) for those with and without MetS respectively (*p* = 0.110). A comparison of ALT (*U* = 17,197.0, *p* = 0.000), AST (*U* = 22,805.0, *p* = 0.015) and ALP (*U* = 21,887.5, *p* = 0.002) mean ranks in participants with and without Mets found that they were all higher in those with MetS.

**Table 3:** Association between cardio-metabolic risk factors and metabolic syndrome

Variable	Categories	Number	Metabolic syndrome status		Univariate		^b^Multivariate	
			Present-No. (%)	Absent-No. (%)	*p*-value	Crude OR (95% CI)	*p*-value	Adjusted OR (95% CI)
Gender	Male^a^	255	64 (25.1%)	191 (74.9%)				
	Female	273	71 (26.0%)	202 (74.0%)	0.811	1.05 (0.71–1.55)	0.523	1.20 (0.68–2.13)
Age (years)	18-29^a^	134	6 (4.5%)	128 (95.5%)				
	30–39	136	23 (16.9%)	113 (83.1%)	0.002	4.34 (1.71–11.04)	0.051	2.64 (1.00–6.99)
	40–49	131	45 (34.4%)	86 (65.6%)	0.000	11.16 (4.56–27.31)	0.000	6.06 (2.35–15.60)
	50–65	127	61 (48.0%)	66 (52.0%)	0.000	19.72 (8.10–48.00)	0.000	11.74 (4.61–29.88)
BMI (kg/m^2^)	<25.00^a^	242	19 (7.9%)	223 (92.1%)				
	25.00–29.99	198	76 (38.4%)	122 (61.6%)	0.000	7.31 (4.22–12.66)	0.000	5.08 (2.80–9.22)
	30.00–39.99	88	40 (45.5%)	48 (54.5%)	0.000	9.78 (5.22–18.34)	0.000	5.23 (2.57–10.66)
WC (cm)	<94 (M) OR <80 (F)^a^	234	4 (1.7%)	230 (98.3%)				
	≥94 (M) OR ≥80 (F)	294	131 (44.6%)	163 (55.4%)	0.000	46.21 (16.75–127.50)		
TGs (mmol/L)	<1.7^a^	437	63 (14.4%)	374 (85.6%)				
	≥1.7	91	72 (79.1%)	19 (20.9%)	0.000	22.50 (12.70–39.85)		
HDL-C (mmol/L)	≥1(M) OR ≥1.3 (F)^a^	304	38 (12.5%)	266 (87.5%)				
	<1(M) OR <1.3 (F)	224	97 (43.3%)	127 (56.7%)	0.000	5.35 (3.48–8.22)		
BP (mmHg)	SBP < 130 OR DBP < 85^a^	287	21 (7.3%)	266 (92.7%)				
	SBP ≥ 130 OR DBP ≥ 85	241	114 (47.3%)	127 (52.7%)	0.000	11.37 (6.82–18.96)		
FPG (mmol/L)	<5.6^a^	445	65 (14.6%)	380 (85.4%)				
	≥5.6	83	70 (84.3%)	13 (15.7%)	0.000	31.48 (16.47–60.16)		
hsCRP (mg/L)	<1.3^a^	262	44 (16.8%)	218 (83.2%)				
	≥1.3	266	91 (34.2%)	175 (65.8%)	0.000	2.58 (1.71–3.89)	0.620	1.14 (0.686–1.880)
GGT (IU/L)	<25^a^	263	45 (17.1%)	218 (82.9%)				
	≥25	265	90 (34.0%)	175 (66.0%)	0.000	2.491 (1.654–3.752)	0.003	2.173 (1.295–3.648)
UA (mmol/L)	<0.31^a^	252	47 (18.7%)	205 (18.3%)				
	≥0.31	276	88 (31.9%)	188 (68.1%)	0.000	2.042 (1.361–3.063)	0.000	2.042 (1.361–3.063)

*Abbreviations BMI* Body Mass Index, *BP* Blood pressure, *CI* Confidence interval, *CVD* Cardiovascular disease, *DBP* Diastolic BP, *F* Female, *FPG* Fasting plasma glucose, *GGT* Gamma-glutamyl transferase, *HDL-C* High density lipoprotein cholesterol, *hs CRP* highly sensitive C-reactive protein, *M* Male, *SBP* Systolic BP, *TGs* Triglycerides, *UA* uric acid, *WC* Waist circumference

^a^Indicates reference category,

^b^Components of MetS excluded in the multivariate analysis

The prevalence of MetS was higher in females though the difference was not statistically significant. There was a statistically significant difference in the prevalence of each component of the MetS when comparing male and female participants except for FPG. The commonest component of the MetS that was present in male participants was elevated BP (53.3%) while in females it was increased WC (71.8%). No female participant had a 10 year CVD risk >10% based on their Framingham risk score compared to 10.6% of males as shown in Table 4.

**Table 4:** Gender comparison of metabolic syndrome component prevalence

Variable	Categories	Male (*n* = 255) No. (%)	Female (*n* = 273) No. (%)	*p*-value
BMI (kg/m^2^)	<25.00	132 (51.8%)	110 (40.3%)	0.001
	25.00–29.99	95 (37.3%)	103 (37.7%)	
	30.00–39.99	28 (11.0%)	60 (22.0%)	
WC (cm)	<94 (M) OR <80 (F)	157 (61.6%)	77 (28.2%)	0.000
	≥94 (M) OR ≥80 (F)	98 (38.4%)	196 (71.8%)	
TGs (mmol/L)	<1.7	194 (76.1%)	243 (89.0%)	0.000
	≥1.7	61 (23.9%)	30 (11.0%)	
HDL-C (mmol/L)	≥1(M) OR ≥1.3 (F)	179 (70.2%)	125 (45.8%)	0.000
	<1(M) OR <1.3 (F)	76 (29.8%)	148 (54.2%)	
BP (mmHg)	SBP < 130 OR DBP < 85	119 (46.7%)	168 (61.5%)	0.001
	SBP ≥ 130 OR DBP ≥ 85	136 (53.3%)	105 (38.5%)	
FPG (mmol/L)	<5.6	212 (83.1%)	233 (85.3%)	0.486
	≥5.6	43 (16.9%)	40 (14.7%)	
10 year CVD risk (%)	≤10%	228 (89.4%)	273 (100.0%)	0.000
	>10%	27 (10.6%)	0 (0.0%)	

*Abbreviations BMI* Body Mass Index, *BP* Blood pressure, *CVD* Cardiovascular disease, *DBP* Diastolic BP, *FPG* Fasting plasma glucose, *F* Female, *HDL-C* High density lipoprotein cholesterol, *M* Male, *SBP* Systolic BP, *TGs* Triglycerides, *WC* Waist circumference

The prevalence of each component of the MetS was significantly higher in those with a BMI greater ≥25 kg/m^2^ compared to those with a BMI < 25 kg/m^2^ as shown in Table 5.

**Table 5:** Comparison of metabolic syndrome component prevalence in different BMI categories

Variable	Categories	BMI < 25.00 (*n* = 242) No. (%)	BMI ≥ 25.00 (*n* = 286) No. (%)	*p*-value
WC (cm)	<94 (M) OR <80 (F)	188 (77.7%)	46 (16.1%)	0.000
	≥94 (M) OR ≥80 (F)	54 (22.3%)	240 (83.9%)	
TGs (mmol/L)	<1.7	223 (92.1%)	214 (74.8%)	0.000
	≥1.7	19 (7.9%)	72 (25.2%)	
HDL-C (mmol/L)	≥1(M) OR ≥1.3 (F)	157 (64.9%)	147 (51.4%)	0.002
	<1(M) OR <1.3 (F)	85 (35.1%)	139 (48.6%)	
BP (mmHg)	SBP < 130 OR DBP < 85	152 (62.8%)	135 (47.2%)	0.000
	SBP ≥ 130 OR DBP ≥ 85	90 (37.2%)	151 (52.8%)	
FPG (mmol/L)	<5.6	230 (95.0%)	215 (75.2%)	0.000
	≥5.6	12 (5.0%)	71 (24.8%)	

*Abbreviations BMI* Body Mass Index, *BP* Blood pressure, *DBP* Diastolic BP, *FPG* Fasting plasma glucose, *F* Female, *HDL-C* High density lipoprotein cholesterol, *M* Male, *SBP* Systolic BP, *TGs* Triglycerides, *WC* Waist circumference

ROC curve analysis of LAP, VAI, WC and BMI ability to predict the presence of MetS showed LAP as having the highest AUC at 0.880. When male and female data were analysed separately, the AUC for the 4 parameters were higher in males with WC, LAP and VAI having excellent AUCs.With the exception of VAI, all other optimum cut-offs were lower in females as shown in Table 6. Using a WC cut-off of 86 cm reduced the prevalence of MetS in female participants from 26.0% to 23.4%.

**Table 6:** Summary of ROC curve and Youden Index analysis for BMI, WC, VAI and LAP as predictors of MetS

Gender	Variable	AUC (95% CI)	YI	Cut-off	Sensitivity	Specificity
Males & Females	BMI	0.766 (0.725–0.808)	0.457	26.23	0.770	0.687
	WC	0.825 (0.789–0.862)	0.518	89.5	0.867	0.651
	VAI	0.858 (0.818–0.897)	0.599	2.057	0.726	0.873
	LAP	0.880 (0.846–0.914)	0.591	37.205	0.815	0.776
Males	BMI	0.841 (0.789–0.892)	0.587	25.71	0.859	0.728
	WC	0.913 (0.879–0.947)	0.759	93.5	0.957	0.806
	VAI	0.905 (0.859–0.951)	0.697	1.727	0.922	0.775
	LAP	0.949 (0.923–0.976)	0.749	42.895	0.843	0.749
Females	BMI	0.702 (0.638–0.765)	0.363	24.99	0.873	0.49
	WC	0.766 (0.710–0.821)	0.453	85.5	0.859	0.594
	VAI	0.814 (0.752–0.876)	0.529	2.065	0.648	0.881
	LAP	0.822 (0.764–0.879)	0.502	30.56	0.775	0.728

*Abbreviations AUC* Area under the curve, *BMI* Body mass index, *CI* Confidence intervals, *LAP* Lipid accumulation product, *WC* Waist circumference, *VAI* Visceral adiposity index, *YI* Youden Index

The lipid parameter that best predictedMetS was TG with AUCs of 0.816, 0.887 and 0.770 for all participants, male and female participants respectively. Measured LDL-C (mLDL-C) was consistently better than calculated LDL-C (cLDL-C) at predicting MetS. Non-HDL-C and cholesterol remnants performed better in males compared to females as shown in Table 7.

**Table 7:** ROC curve analysis for lipid parameters as predictors of MetS

Gender	Variable	AUC (95% CI)	YI	Cut-off	Sensitivity	Specificity
Males & Females	TG	0.816 (0.769–0.862)	0.517	1.27	0.726	0.791
	HDL-C	0.721 (0.669–0.774)	0.318	1.1	0.756	0.562
	mLDL-C	0.660 (0.605–0.714)	0.268	3.0	0.652	0.616
	cLDL-C	0.578 (0.520–0.636)	0.146	3.2	0.459	0.687
	TC	0.648 (0.592–0.704)	0.258	5.0	0.548	0.710
	non HDL-C	0.706 (0.654–0.759)	0.342	3.4	0.785	0.557
	CholRem	0.751 (0.701–0.801)	0.421	0.7	0.622	0.799
Males	TG	0.887 (0.841–0.933)	0.645	1.68	0.734	0.911
	HDL-C	0.722 (0.642–0.803)	0.395	0.9	0.594	0.801
	mLDL-C	0.719 (0.650–0.789)	0.346	2.8	0.828	0.518
	cLDL-C	0.613 (0.532–0.693)	0.190	2.8	0.703	0.487
	TC	0.731 (0.660–0.802)	0.358	4.9	0.688	0.670
	non HDL-C	0.778 (0.714–0.843)	0.456	3.5	0.891	0.565
	CholRem	0.814 (0.748–0.879)	0.551	0.7	0.813	0.738
Females	TG	0.770 (0.697–0.843)	0.471	1.25	0.620	0.851
	HDL-C	0.752 (0.684–0.820)	0.412	1.1	0.704	0.708
	mLDL-C	0.605 (0.525–0.685)	0.214	3.1	0.521	0.693
	cLDL-C	0.547 (0.464–0.629)	0.123	3.5	0.296	0.827
	TC	0.572 (0.490–0.654)	0.173	5.0	0.465	0.708
	non HDL-C	0.645 (0.567–0.722)	0.264	3.4	0.690	0.574
	CholRem	0.712 (0.642–0.782)	0.307	0.7	0.451	0.856

*Abbreviations AUC* Area under the curve, *CholRem* Cholesterol remnants, *cLDL-C* calculated LDL cholesterol, *CI* Confidence intervals, *mLDL-C* measured LDL cholesterol, *non HCL-C* non HDL cholesterol, *TC* Total cholesterol, *TG* Triglycerides, *YI* Youden Index

## Discussion

The prevalence of MetS of 25.6% found in this study is quite high given the strict inclusion and exclusion criteria used for the RI study. This sample was meant to be representative of a healthy black African population in Kenya and as such the percentage of individuals with MetS was expected to be much lower. Several studies have reported the prevalence of MetS in Africa. These studies are quite heterogeneous by way of individuals included and MetS definitions used hence limiting direct comparisons. Ogbera reported a MetS prevalence of 86% using the 2009 harmonized definition in diabetic patients in Lagos, Nigeria [22]. This high prevalence in a diabetic population is expected given the known association between MetS and increased risk of developing type 2 diabetes [23]. In Ethiopia, Tran et al. reported a prevalence of 12.5% and 17.9% using the ATP III and IDF definitions respectively in a presumably healthy population comprising working class individuals in Addis Ababa [24]. This prevalence is much lower than what we have found in our study. Of, note is that the prevalence of overweight and obese individuals was lower in the Ethiopian population which we hypothesize may be attributed to genetic and/or environmental differences. Kaduka et al. reported a prevalence of 34.6% in an urban population in Nairobi, Kenya. This study was however limited to one constituency in Nairobi hence limiting the generalizability of the findings. The inclusion criteria was also not as strict as what we used in our study and this could partly explain the higher reported prevalence [20].

The most prevalent component of the MetS was increased WC which was present in 294 (55.7%) participants followed by elevated BP found in 241 (45.6%) of study participants. This is consistent with what has been found in other studies carried out in sub-Saharan Africa (SSA) [20, 24]. However, the most common MetS component in males was hypertension (53.3%) and increased WC (71.8%) in females. In South Africa, HDL-C < 1.3 mmol/L was found to be the most prevalent of the MetS components in black African women at 70.1% closely followed by increased WC at 69.3% compared to 54.2% and 71.8% for low HDL-C and increased WC respectively in our study [25]. WC is a measure of both VAT and SAT. Increased visceral adiposity contributes to the insulin resistance that is central in the pathogenesis of MetS and is associated with the production of adipocytokines which contribute to the chronic low grade inflammation seen in MetS. WC was a better predictor of MetS than BMI in our study but had a lower AUC than both LAP and VAI. Chen et al. found that VAI was better than BMI and WC in predicting the presence of diabetes in a Chinese cohort [16]. Gender specific ROC curve analysis in our study showed that WC was a better predictor of MetS in males than in females and was superior to VAI. A WC greater than 93.5 cm in male participants had a sensitivity of 95.7%, specificity of 80.6% and AUC of 0.913 which is excellent for a simple measurement that can be carried out in any clinical setting. It is interesting that the optimum WC cut-off of 93.5 cm is similar to the proposed European cut-off for abdominal obesity of 94 cm that is used in the MetS 2009 harmonized criterion for men. The women cut-off of 85.5 cm is much higher than the European cut-off of 80 cm highlighting the need to adopt population specific cut-offs [4]. The use of the 80 cm cut-off in black African women may be misclassifying many of them as having abdominal obesity. A study carried out in Johannesburg, South Africa derived an optimal cut-off to diagnose MetS in urban black African women of 91.5 cm. The prevalence of MetS in this population was 42.1% and the WC was measured at the smallest girth above the umbilicus [25]. This suggests that the optimal cut-off would have been higher if measured at the level of the umbilicus like we did in our study. Further longitudinal studies are required to validate the WC cut-offs we have proposed and assess their ability to predict CVD risk in a black African population.

Elevated GGT and UA levels independently more than doubled the odds of having MetS. Surprisingly, elevated hsCRP did not independently increase the odds of one having MetS. Elevated GGT is known to occur in non-alcoholic fatty liver disease (NAFLD) which represents the hepatic component of MetS and its increase may be a physiological response to the increased oxidation stress seen in MetS as GGT plays a role in the metabolism of glutathione which is an important anti-oxidant [26]. Matsha et al. demonstrated that increasing levels of GGT were associated with increasing levels of insulin resistance as well as an increase in the number of components of MetS present in a mixed ancestry population in South Africa. This was so even in GGT levels within the reference interval. However, additional tests of liver enzymes were not performed in this study to enable inferences on the pathophysiology behind the increase of GGT in MetS [27]. In our study, elevated GGT was associated with an increase in ALT, AST and ALP suggesting that MetS may be associated with mild hepatitis and cholestasis. It is known that hepatocyte injury does occur in individuals with NAFLD, however, canalicular cholestasis is more common in alcoholic liver disease [28].

Despite the difference in prevalence of Mets in male and female participants not being statistically significant, the 10 year CVD risk was significantly higher in male participants with 10.6% having a greater than 10% risk compared to 0% among females. This suggests that the presence of MetS confers a different CVD risk depending on gender with the risk being lower in females. We explored the possibility of over diagnosis of MetS in female participants given the disparity in WC between the Caucasian cut-off and the higher values for females reported in studies carried out in Africa including our study. However, whereas using a WC cut-off of 86 cm reduced the MetS prevalence in females from 26.0% to 23.4%, the difference compared to males was still not statistically significant hence this doesn’t explain the observed disparity in CVD risk. This is even more surprising when you consider that women had a statistically significant higher level of hsCRP than men. This finding further adds to the uncertainty surrounding the use of hsCRP in CVD risk prediction [29]. The optimal cut-off for TGs that predicted the presence of MetS was 1.27 mmol/L for combined males and females, 1.68 mmol/L for males and 1.25 mmol/L for females. Given these findings, we do advocate the adoption of separate TG cut-offs for males and females for the diagnosis of MetS in a black African population. The higher TG levels and WC in men compared to women is in keeping with the known positive correlation between TG levels and visceral adiposity [30]. The MetS 2009 harmonized criterion uses a cut-off of 1.7 mmol/L for both males and females which could under estimate the prevalence of hypertriglyceridaemia in female black Africans and by extension MetS. Using gender specific TG cut-offs in addition to adjusting the WC cut-off to 86 cm increased the MetS prevalence in females to 29.7%. The prevalence in males remained at 25.1% which is not surprising given the similarities in our male TG and WC cut-offs to those used in the harmonized criterion [4].

Of the lipid parameters that are not included in the MetS definition, cholesterol remnants outperformed non-HDL-C, mLDL-C and cLDL-C in predicting the presence of MetS. Calculated non-fasting cholesterol remnant has been shown to be an independent risk factor for ischaemic heart disease and myocardial infarction in a Caucasian population [17, 18]. There is a paucity of data on the utility of cholesterol remnants and non HDL-C in predicting the presence of MetS in black Africans. In our study, the lipid parameters were better predictors in males than in females. Prospective cohort studies are needed to determine whether cholesterol remnants are an independent risk factor for CVD in black Africans.

This study has several limitations. It is a cross sectional study hence causal inferences cannot be made. The predictive ability of the factors analysed would best be studied in a longitudinal study and as such, our findings would need to be validated in a prospective cohort study. However, with a sample size of 528, the study has sufficient power to draw conclusions on optimal cut-offs for MetS prediction. We also didn’t collect data on family history of CVD and therefore our estimation of CVD risk based on Framingham calculations may have underestimated the 10 year risk. However, it is unlikely that this omission significantly affected the comparison between CVD risk in males and females. Our study was carried out in an urban population hence the findings cannot be directly extrapolated to a rural population who are known to have a lower prevalence of obesity and MetS.

## Conclusion

We propose 94 cm and 86 cm as WC cut-offs for males and females respectively for black Africans in Kenya when diagnosing MetS. These however need to be validated prospectively in other countries in SSA. There is also a need to adopt gender specific MetS TG cut-offs to avoid over diagnosing hypertriglyceridaemia in black African women who have a lower TG level compared to the 1.7 mmol/L cut-off. The high prevalence of abdominal obesity, hypertension and MetS in our subjectively healthy population emphasizes the need for primary healthcare interventions to control the epidemic of CVD in Kenya. Urbanization in SSA will continue to accelerate the incidence of obesity, diabetes and hypertension all of which can be avoided or at least reduced by adopting lifestyle changes such as reduced intake of processed food that is high in calories and regular exercise to ensure a reasonable balance between what is consumed and what is expended. Unless urgent interventions are put in place to prevent this epidemic, the additional morbidity due to CVD will over burden the already stretched healthcare services in SSA leading to increased mortality.

## Abbreviations

ALP: Alkaline phosphatase
ALT: Alanine aminotransferase
AST: Aspartate aminotransferase
ATP III: Adult treatment panel III
AUC: Area under the curve
BMI: Body mass index
BP: Blood pressure
CholRem: Cholesterol remnants
CI: Confidence interval
cLDL-C: Calculated low density lipoprotein cholesterol
C-RIDL: Committee of reference intervals and decision limits
CT: Computed tomography
CVD: Cardiovascular disease
FPG: Fasting plasma glucose
GGT: Gamma glutamyl transferase
HDL-C: High density lipoprotein cholesterol
hsCRP: Highly sensitive c-reactive protein
IDF: International diabetes federation
IDL: Intermediate density lipoprotein
IFCC: International Federation of Clinical Chemistry
IQR: Interquartile range
ISO: International Organization of Standards
LAP: Lipid accumulation product
LDL-C: Low density lipoprotein cholesterol
MetS: Metabolic syndrome
mLDL-C: Measured low density lipoprotein cholesterol
MRI: Magnetic resonance imaging
NCD: Non communicable disease
OR: Odds ratio
ROC: Receiver operator curves
SAT: Subcutaneous adipose tissue
SSA: Sub -Saharan Africa
TC: Total cholesterol
TG: Triglyceride
UA: Uric acid
VAI: Visceral adiposity index
VAT: Visceral adipose tissue
VLDL: Very low density lipoprotein
WC: Waist circumference
YI: Youden index

## References

[1] Mathers CD, Loncar D (2006). Projections of global mortality and burden of disease from 2002 to 2030. PLoS Med.

[2] Steyn K, Sliwa K, Hawken S, Commerford P, Onen C, Damasceno A, Ounpuu S, Yusuf S, INTERHEART Investigators in Africa (2005). Risk factors associated with myocardial infarction in Africa: the INTERHEART Africa study. Circulation.

[3] Vorster HH (2002). The emergence of cardiovascular disease during urbanisation of Africans. Public Health Nutr.

[4] Alberti KGMM, Eckel RH, Grundy SM, Zimmet PZ, Cleeman JI, Donato KA, Fruchart J-C, James WPT, Loria CM, Smith SC (2009). Harmonizing the metabolic syndrome: a joint interim statement of the International Diabetes Federation Task Force on Epidemiology and Prevention; National Heart, Lung, and Blood Institute; American Heart Association; World Heart Federation; International. Circulation.

[5] Gami AS, Witt BJ, Howard DE, Erwin PJ, Gami LA, Somers VK, Montori VM (2007). Metabolic syndrome and risk of incident cardiovascular events and death: a systematic review and meta-analysis of longitudinal studies. J Am Coll Cardiol.

[6] Wildman RP, Muntner P, Reynolds K, McGinn AP, Rajpathak S, Wylie-Rosett J, Sowers MR (2008). The Obese Without Cardiometabolic Risk Factor Clustering and the Normal Weight With Cardiometabolic Risk Factor Clustering. Arch Intern Med.

[7] Ichihara K (2014). Statistical considerations for harmonization of the global multicenter study on reference values. Clin Chim Acta.

[8] Reilly MP (2003). The Metabolic Syndrome: More Than the Sum of Its Parts?. Circulation.

[9] Shah RV, Murthy VL, Abbasi SA, Blankstein R, Kwong RY, Goldfine AB, Jerosch-Herold M, Lima JAC, Ding J, Allison MA (2014). Visceral Adiposity and the Risk of Metabolic Syndrome Across Body Mass Index: The MESA Study. JACC Cardiovasc Imaging.

[10] Kahn HS (2005). The &quot;lipid accumulation product&quot; performs better than the body mass index for recognizing cardiovascular risk: a population-based comparison. BMC Cardiovasc Disord.

[11] Taverna MJ, Martinez-Larrad MT, Frechtel GD, Serrano-Rios M (2011). Lipid accumulation product: a powerful marker of metabolic syndrome in healthy population. Eur J Endocrinol.

[12] Tellechea ML, Aranguren F, Martínez-Larrad MT, Serrano-Ríos M, Taverna MJ, Frechtel GD: Ability of Lipid Accumulation Product to Identify Metabolic Syndrome in Healthy Men From Buenos Aires. Diabetes Care. 2009;32(7):e85–e85. 10.2337/dc08-2284 19564464

[13] Chiang J-K, Koo M (2012). Lipid accumulation product: a simple and accurate index for predicting metabolic syndrome in Taiwanese people aged 50 and over. BMC Cardiovasc Disord.

[14] Wakabayashi I, Daimon T (2014). A strong association between lipid accumulation product and diabetes mellitus in japanese women and men. J Atheroscler Thromb.

[15] Amato MC, Giordano C, Galia M, Criscimanna A, Vitabile S, Midiri M, Galluzzo A, AlkaMeSy Study Group (2010). Visceral Adiposity Index: A reliable indicator of visceral fat function associated with cardiometabolic risk. Diabetes Care.

[16] Chen C, Xu Y, Guo Z, Yang J, Wu M, Hu X (2014). The application of visceral adiposity index in identifying type 2 diabetes risks based on a prospective cohort in China. Lipids Health Dis.

[17] Jorgensen AB, Frikke-Schmidt R, West AS, Grande P, Nordestgaard BG, Tybjaerg-Hansen A (2013). Genetically elevated non-fasting triglycerides and calculated remnant cholesterol as causal risk factors for myocardial infarction. Eur Heart J.

[18] Varbo A, Benn M, Tybjærg-Hansen A, Jørgensen AB, Frikke-Schmidt R, Nordestgaard BG (2013). Remnant Cholesterol as a Causal Risk Factor for Ischemic Heart Disease. J Am Coll Cardiol.

[19] Gyakobo M, Amoah AG, Martey-Marbell D-A, Snow RC (2012). Prevalence of the metabolic syndrome in a rural population in Ghana. BMC Endocr Disord.

[20] Kaduka LU, Kombe Y, Kenya E, Kuria E, Bore JK, Bukania ZN, Mwangi M (2012). Prevalence of metabolic syndrome among an urban population in Kenya. Diabetes Care.

[21] Blom D (2011). Cardiovascular risk assessment. Medpharm S Afr Fam Pr.

[22] Ogbera AO (2010). Prevalence and gender distribution of the metabolic syndrome. Diabetol Metab Syndr.

[23] Marott SCW, Nordestgaard BG, Tybjærg-Hansen A, Benn M (2016). Components of the Metabolic Syndrome and Risk of Type 2 Diabetes. J Clin Endocrinol Metab.

[24] Tran A, Gelaye B, Girma B, Lemma S, Berhane Y, Bekele T, Khali A, Williams MA (2011). Prevalence of Metabolic Syndrome among Working Adults in Ethiopia. Int J Hypertens.

[25] Crowther NJ, Norris SA (2012). The current waist circumference cut point used for the diagnosis of metabolic syndrome in sub-Saharan African women is not appropriate. PLoS One.

[26] Lee D-H, Blomhoff R, Jacobs DR (2004). Is serum gamma glutamyltransferase a marker of oxidative stress?. Free Radic Res.

[27] Matsha TE, Macharia M, Yako YY, Erasmus RT, Hassan MS, Kengne AP (2014). Gamma-glutamyltransferase, insulin resistance and cardiometabolic risk profile in a middle-aged African population. Eur J Prev Cardiol.

[28] Takahashi Y, Fukusato T (2014). Histopathology of nonalcoholic fatty liver disease/nonalcoholic steatohepatitis. World J Gastroenterol.

[29] Yousuf O, Mohanty BD, Martin SS, Joshi PH, Blaha MJ, Nasir K, Blumenthal RS, Budoff MJ (2013). High-Sensitivity C-Reactive Protein and Cardiovascular Disease: A Resolute Belief or an Elusive Link?. J Am Coll Cardiol.

[30] Taniguchi A, Nakai Y, Sakai M, Yoshii S, Hamanaka D, Hatae Y, Kawata M, Yamanouchi K, Okumura T, Doi K, Tokuyama K, Nagasaka S, Fukushima M (2002). Relationship of regional adiposity to insulin resistance and serum triglyceride levels in nonobese Japanese type 2 diabetic patients. Metabolism.

