# Erratum to: Metabolic syndrome and its predictors in an urban population in Kenya: a cross sectional study

**DOI:** 10.1186/s12902-017-0195-1

**Published:** 2017-07-14

**Authors:** Geoffrey Omuse, Daniel Maina, Mariza Hoffman, Jane Mwangi, Caroline Wambua, Elizabeth Kagotho, Angela Amayo, Peter Ojwang, Zulfiqarali Premji, Kiyoshi Ichihara, Rajiv Erasmus

**Affiliations:** 1 Department of Pathology, Aga Khan University Hospital Nairobi, P.O. Box 30270-00100, Nairobi, Kenya; 2 Division of Chemical Pathology, Department of Pathology, Stellenbosch University, Tygerberg Hospital, P.O. Box 19113, Cape Town, South Africa; 3 PathCare Kenya Ltd, P.O. Box 12560-00606, Nairobi, Kenya; 4 Department of Human Pathology, University of Nairobi, P.O. Box 19676-00200, Nairobi, Kenya; 5 Department of Pathology, Maseno University, P.O. Box Private Bag, Maseno, Kenya; 6 Formerly of Muhimbili University of Health and Allied Sciences, Dar es Salaam, Tanzania; 7 Graduate School of Medicine, Faculty of Health Sciences, Yamaguchi University, Minami-Kogushi 1-1-1, Ube, 755-8505 Japan

## Erratum

The original article [1] was updated to correct a mistake in the third sentence of the conclusions section:

*‘There is also a need to adopt gender specific MetS TG cut-offs to avoid* ***over*** *diagnosing hypertriglyceridaemia in black African women who have a lower TG level compared to the 1.7 mmol/L cut-off.’*

Has been changed to:

*‘There is also a need to adopt gender specific MetS TG cut-offs to avoid* ***under*** *diagnosing hypertriglyceridaemia in black African women who have a lower TG level compared to the 1.7 mmol/L cut-off.’*
